# Protective Effect of N-Acetyl-Cysteine (NAC) in Lipopolysaccharide (LPS)-Associated Inflammatory Response in Rat Neonates

**DOI:** 10.5041/RMMJ.10303

**Published:** 2017-04-28

**Authors:** Nizar Khatib, Zeev Weiner, Yuval Ginsberg, Nibal Awad, Ron Beloosesky

**Affiliations:** 1 Department of Obstetrics and Gynecology, Rambam Health Care Campus, Haifa, Israel; 2 Bruce Rappaport Faculty of Medicine, Technion–Israel Institute of Technology, Haifa, Israel

**Keywords:** Infection, lipopolysaccharide, N-acetyl-cysteine, neonatal period

## Abstract

**Objective:**

Increased inflammatory response may be associated with adverse clinical outcomes, especially in the neonatal period. The aims of this study were to determine whether N-acetyl-cysteine (NAC), an anti-inflammatory agent, attenuates the inflammatory response in young rats and to determine the most effective route of administration.

**Methods:**

Four groups of Sprague-Dawley rats (in each group four rats) were studied at 30 days of age. One hour following intraperitoneal (IP) injection of lipopolysaccharide 50 μg/kg, the rats were randomized to subcutaneous (SC), per os (PO), or intraperitoneal (IP) injection of NAC 300 mg/kg, or saline. The control group received saline injection (IP). Three hours following the N-acetyl-cysteine injection the rats were sacrificed, then serum tumor necrosis factor-α (TNF-α) and IL-6 levels were determined by ELISA.

**Results:**

Lipopolysaccharide significantly increased the neonatal serum IL-6 and TNF-α (2051.0±349 and 147.0±25.8 pg/mL, respectively; *P<*0.01) levels compared to 10 pg/mL in the controls. N-acetyl-cysteine administered one hour following lipopolysaccharide injection significantly attenuated the inflammatory response. Intraperitoneal administration of NAC decreased IL-6 and TNF-α concentration to 294.6 and 17.1 pg/mL, respectively, and was more effective than SC or PO administration.

**Conclusions:**

N-acetyl-cysteine attenuated the inflammatory response in the neonatal rats, and IP was the most effective administration route.

## INTRODUCTION

The inflammatory response to infection is the host’s most important initial defense mechanism against pathogens. An imbalance between the pro- and anti-inflammatory responses may have adverse effects on the host. Severe postnatal infections lead to a systemic inflammatory response with excessive release of cytokines (interleukin-1 [IL-1], IL-8, and tumor necrosis factor [TNF]-α), which can be a stressful event for the newborn. Inflammation is associated with the generation of a large quantity and variety of free radicals and reactive oxygen species (FR/ROS) that increase the production and secretion of the cytokines.^1^ In the neonatal period, infants are more vulnerable to infections. In many cases, tissue damage associated with the infection is a consequence of increased proinflammatory response rather than directly from the bacteria.^2^ There is imbalance between the pro- and anti-inflammatory responses in newborns and neonates that may further increase the risk of infection in early life. Each year more than a million infants are estimated to die in their first four weeks from infections, and Gram-negative rods account for the majority of deaths.^3^

The adverse effect of neonatal infection is not limited to the neonatal period and may extend into adulthood. There is intriguing evidence that some central nervous system (CNS)-mediated immune responses may be influenced by early neonatal stress. Bilbo et al.^4^ found that neonatal exposure to bacteria (*Escherichia coli*) in rats is associated with memory impairment in adulthood, thus neonatal infection appears to create a state of “vulnerability” that extends into adulthood. This may lead to an elevation of glial cell markers and induce exaggerated IL-1β brain response in adulthood, which appears to underlie memory impairment.^5^

Attenuating the immune response may decrease the risk associated with severe inflammatory response. N-acetyl-cysteine (NAC) is a known antioxidant and anti-inflammatory agent that has been shown to ameliorate the inflammatory response by blocking nuclear factor (NF)-κb activation. Several studies reported that NAC has a protective effect following lipopolysaccharide (LPS)-induced inflammation during the prenatal period.^6^–^8^ Treatment with NAC significantly reduced neonatal brain injury associated with maternal LPS injection and attenuated maternal and amniotic fluid oxidative stress even when administered after lipopolysaccharide injection.^6^–^8^ In this study, we sought to determine whether NAC can attenuate the inflammatory response associated with LPS stimulation in the neonatal period and to explore the preferred route of administration.

## METHODS

### Animals and Treatments

Five groups of Sprague-Dawley rats (Harlan Sprague-Dawley) were studied at age 30 days (four rats in each group). The rats were maintained in temperature (37°C) and light (06.00, lights on; 18.00, lights off) controlled facilities with access to food (Laboratory Rodent Diet 5001; LabDiet, St Louis, MO, USA), and provided water ad libitum. Lipopolysaccharide was administered intraperitoneally. Lipopolysaccharide (*E. coli* serotype 0111; B4, Calbiochem; Merck, Darmstadt, Germany) was reconstituted in physiological saline and administered at 50 μg/kg. N-acetyl-cysteine (Sigma, St Louis, MO, USA) was reconstituted in physiological saline at pH 6.8–7.2 and administered at 300 mg/kg per os (PO) or using intraperitoneal (IP) or subcutaneous (SC) injection. The first group (control) received IP saline injection one hour following the first saline injection, the second group received IP saline injection one hour following the LPS injection, the third group received IP NAC one hour following the LPS injection, the fourth group received PO NAC one hour following the LPS injection, and the fifth group received SC NAC one hour following the LPS injection.

The protocols and procedures for this study were approved by the Institutional Animal Care and Utilization Committee (IACUC) at The Rappaport Family Institute for Research in the Medical Sciences.

### Sample Collection

Three hours following LPS injections, the rats were anesthetized with pentobarbital, and their hearts were exposed via midline incision. Blood was collected via cardiac puncture and centrifuged at 4°C to isolate serum. All samples were subsequently stored at −80°C for further processing and analyses. All samples were analyzed individually.

### Statistical Methods

Statistical analysis was performed using SPSS version 21 (SPSS, Inc., Chicago, IL, USA). The normality of data was tested by the Kolmogorov–Smirnov test, and one-way analysis of variances (ANOVA) was used to analyze the differences in the protein expression between the five treatment groups. Differences were considered to be significant at *P<*0.05.

## RESULTS

**Figure 1:**
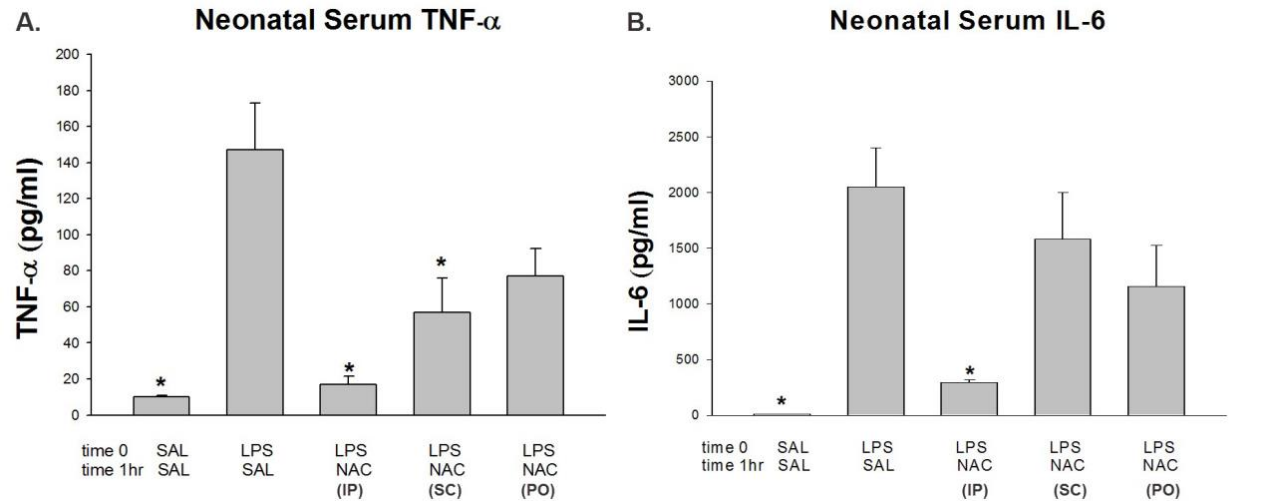
Levels of Serum TNF-α and Serum IL-6 in Rat Neonates. **(A)** Serum TNF-α. (**B)** Serum IL-6. SAL-SAL received two IP saline injections; LPS-SAL received IP LPS followed by IP SAL; LPS-NAC (IP) received IP NAC one hour following the LPS injection; LPS-NAC (SC) received SC NAC one hour following the LPS injection; LPS-NAC (PO) received PO NAC one hour following the LPS injection. *P<0.05, significantly different from the LPS-SAL group. IP, intraperitoneal; LPS, lipopolysaccharide; NAC, N-acetyl-cysteine; PO, per os; SAL, saline; SC, subcutaneous.

Lipopolysaccharide induced a significant increase in the neonatal serum IL-6 and TNF-α (2051.0±349 and 147.0±25.8 pg/mL, respectively; *P<*0.01) compared to control 10 pg/mL. N-acetyl-cysteine administered IP one hour following LPS injection significantly attenuated both IL-6 and TNF-α serum levels (294.6±23 and 17.1±4.5 pg/mL respectively; *P<*0.01). N-acetyl-cysteine administered SC was less effective: it decreased significantly only TNF-α (56.8±19.3 pg/mL; *P<*0.05), with no change in IL-6 levels. *Per os* administration of NAC was the least effective way to decrease TNF-α to near significant levels (77.0±15.5 pg/mL; *P=*0.059), with no change in IL-6 levels (Figure 1).

## DISCUSSION

Lipopolysaccharide significantly increased the neonatal inflammatory response in serum compared to controls. Administration of NAC one hour following LPS injection significantly attenuated the inflammatory response when administered IP, and only partly when administered PO or SC.

Early exposure to infectious agents may have significant consequences to a neonate, leading to death or significantly impacting the development and function of physiological systems throughout an individual’s life. Sepsis is one of the leading causes of death in hospitalized neonates and preterm infants.^9^ Every year, over 1 million neonates worldwide die within their first weeks of life due to neonatal sepsis.^3^ The mortality rate associated with neonatal sepsis is higher in neonates than in children or adults.^10^,^11^ The tissue damage in many cases of infection is associated with the severe inflammatory response rather than with the infection. In Gram-negative bacteria, LPS from the bacterial wall initiates the inflammatory response.

Attenuating the inflammatory response may protect the neonate and have a synergistic effect with antibiotics treatment. While antibiotics may kill the bacteria, the inflammatory cascade can continue to damage tissue. N-acetyl-cysteine attenuates inflammatory responses via its antioxidant properties; *in vivo* NAC is converted into metabolites capable of stimulating glutathione (GSH) synthesis, promoting detoxification, and acts directly as free radical scavengers.^12^,^13^ In humans, administration of NAC decreased NF-κb activation associated with a decrease in IL-8 in septic patients.^14^ It is intriguing that NAC administered one hour post LPS suppressed the inflammatory response in the neonate serum; as we and others^4^,^15^ have previously shown, proinflammatory cytokine TNF-α induction following LPS treatment reaches maximal levels in the serum within one hour and returns to control levels in 12 hours. This inhibitory effect indicates that NAC can attenuate and suppress the inflammatory response even if injected after the process has been initiated.^8^

In our study, we administered NAC through different routes. The attenuated inflammatory response was evident in all three administration groups (IP, PO, and SC); however, the most significant response was demonstrated in the IP group. In previous studies, NAC was administered as an antioxidant and anti-inflammatory in animal models through different routes and in different clinical scenarios. Wang et al.^16^ demonstrated that IP NAC administration was neuroprotective in eight-day-old neonatal rats in an ischemia-reperfusion model. Buhimschi et al.^14^ reported restoration of maternal and fetal oxidative balance after oral administration of NAC in an LPS model; SC administration of NAC was also reported to be effective.^17^ The difference in the study’s protocols, routes of administration, and dosages of NAC makes it difficult to evaluate the preferred mode of delivery in the animal models. N-acetyl-cysteine is being studied in many clinical scenarios in rodents, as finding the best route of administration will inform the best methodology and validate experimental results. In this study, we demonstrated that IP administration of NAC was the superior method in attenuating neonatal inflammatory response, and IP NAC significantly decreased serum IL-6 and TNF-α levels. Intraperitoneal administration may bypass the liver, which is responsible for elimination of the drug and thus allows for higher levels of NAC to remain in the blood.

Our findings provide novel insights into the role of NAC in attenuating the inflammatory response in neonatal rats. The effectiveness of NAC is not limited to the neonatal period; previous studies reported that NAC administration is effective in treating infections and respiratory complications in adults.^18^–^20^ To the best of our knowledge, this is the first study that addresses the question of the most effective route of NAC administration in a neonatal inflammatory rodent model. In light of the short- and long-term complications associated with neonatal infection, we hypothesize that during the “vulnerability” state antibiotics alone cannot reduce or inhibit an inflammatory process already underway. Our results suggest that NAC is a candidate agent that may be of therapeutic value in conditions associated with neonatal infection. Treatment with NAC, in addition to antibiotics, may help restore the oxidative balance, as evident in our study. In summary, we have demonstrated that, in a rat model, LPS-induced neonatal cytokine responses can be attenuated most effectively by IP NAC administration. Further research is needed to investigate the effectiveness of combining IP NAC with antibiotics in treating neonatal infection.

## Abbreviations

CNS: central nervous system
FR/ROS: free radicals/reactive oxygen species
GSH: glutathione
IACUC: Institutional Animal Care and Utilization Committee
IP: intraperitoneal
LPS: lipopolysaccharide
NAC: N-acetyl-cysteine
PO: per os
SC: subcutaneous
TNF-α: tumor necrosis factor-α

## References

[1] Balbior BM (1995). The respiratory burst oxidase. Curr Opin Hematol.

[2] Gustot T (2011). Multiple organ failure in sepsis: prognosis and role of systemic inflammatory response. Curr Opin Crit Care.

[3] Lawn JE, Cousens S, Zupan J (2005). 4 million neonatal deaths: When? Where? Why?. Lancet.

[4] Bilbo SD, Levkoff LH, Mahoney JH (2005). Neonatal infection induces memory impairments following an immune challenge in adulthood. Behav Neurosci.

[5] Bilbo SD, Biedenkapp JC, Der-Avakian A, Watkins LR, Rudy JW, Maier SF (2005). Neonatal infection-induced memory impairment after lipopolysaccharide in adulthood is prevented via caspase-1 inhibition. J Neurosci.

[6] Beloosesky R, Weiner Z, Ginsberg Y, Ross MG (2012). Maternal N-acetyl-cysteine (NAC) protects the rat fetal brain from inflammatory cytokine responses to lipopolysaccharide (LPS). J Matern Fetal Neonatal Med.

[7] Beloosesky R, Ginsberg Y, Khatib N (2013). Prophylactic maternal N-acetylcysteine in rats prevents maternal inflammation-induced offspring cerebral injury shown on magnetic resonance imaging. Am J Obstet Gynecol.

[8] Awad N, Khatib N, Ginsberg Y (2011). N-acetyl-cysteine (NAC) attenuates LPS-induced maternal and amniotic fluid oxidative stress and inflammatory responses in the preterm gestation. Am J Obstet Gynecol.

[9] Meadow W, Frain L, Ren Y, Lee G, Soneji S, Lantos J (2002). Serial assessment of mortality in the neonatal intensive care unit by algorithm and intuition: certainty, uncertainty, and informed consent. Pediatrics.

[10] Barton P, Kalil AC, Nadel S (2004). Safety pharmacokinetics, and pharmacodynamics of drotrecogin alfa (activated) in children with severe sepsis. Pediatrics.

[11] Martin GS, Mannino DM, Eaton S, Moss M (2003). The epidemiology of sepsis in the United States from 1979 through 2000. N Engl J Med.

[12] Flanagan RJ, Meredith TJ Use of N-acetylcysteine in clinical toxicology. Am J Med.

[13] Harrison PM, Wendon JA, Gimson AE, Alexander GJ, Williams R (1991). Improvement by acetylcysteine of hemodynamics and oxygen transport in fulminant hepatic failure. N Engl J Med.

[14] Buhimschi IA, Buhimschi CS, Weiner CP (2003). Protective effect of N-acetylcysteine against fetal death and preterm labor induced by maternal inflammation. Am J Obstet Gynecol.

[15] Urakubo A, Jarskog LF, Liebeman JA, Gilmore JH (2001). Prenatal exposure to maternal infection alters cytokine expression in the placenta, amniotic fluid, and fetal brain. Schizophr Res.

[16] Wang X, Svedin P, Nie C (2007). N-Acetylcysteine reduces lipopolysaccharide-sensitized hypoxic-ischemic brain injury. Ann Neurol.

[17] Tsunevoshi H, Akatsuka N, Ohno M, Hara K, Ochiai M, Moroi M (1989). Inhibition of development of tolerance to nitroglycerin by preventive administration of N-acetylcysteine in rats. Jpn Heart J.

[18] Aslam S, Trautner BW, Ramanathan V, Darouiche RO (2008). Pilot trial of N-acetylsteine and tigecycline as a catheter-lock solution for treatment of hemodialysis catheter bacteremia. Infect Control Hosp Epidemiol.

[19] Schaller G, Pleiner J, Mittermaver F, Posch M, Kapiotis M, Wolzt M (2007). Effects of N-acetylcysteine against systemic and renal hemodynamic effects of endotoxin in healthy humans. Crit Care Med.

[20] Zingg U, Hofer CK, Seifert B, Metzger U, Zollinger A (2007). High dose N-acetylcysteine to prevent pulmonary complications in partial or total transthoracic esophagectomy: results of a prospective observational study. Dis Esophagus.

